# Metacognitive Therapy for Depression: A 3-Year Follow-Up Study Assessing Recovery, Relapse, Work Force Participation, and Quality of Life

**DOI:** 10.3389/fpsyg.2019.02908

**Published:** 2019-12-23

**Authors:** Stian Solem, Leif Edward Ottesen Kennair, Roger Hagen, Audun Havnen, Hans M. Nordahl, Adrian Wells, Odin Hjemdal

**Affiliations:** ^1^Department of Psychology, Norwegian University of Science and Technology, Trondheim, Norway; ^2^Department of Mental Health, Norwegian University of Science and Technology (NTNU), Trondheim, Norway; ^3^Division of Psychiatry, St. Olavs Hospital, Nidaros Distriktspsykiatriske Senter (DPS), Trondheim, Norway; ^4^Faculty of Biology, Medicine and Health, School of Psychological Sciences, Manchester Academic Health Science Centre, The University of Manchester, Manchester, United Kingdom; ^5^Greater Manchester Mental Health NHS Trust, Prestwich, United Kingdom

**Keywords:** depression, metacognitive therapy, metacognition, long-term follow-up, recovery, relapse prevention, quality of life

## Abstract

A major challenge in the treatment of depression has been high relapse rates following treatment. The current study reports results from a 3-year follow-up of patients treated with metacognitive therapy (MCT). Thirty-four of the 39 patients enrolled in the original study attended assessment (participation rate of 87%). There were large reductions in symptoms of depression, anxiety, interpersonal problems, and worry, as well as metacognitive beliefs. Three patients fulfilled diagnostic criteria for axis-I disorders: one with depression and two with generalized anxiety disorder. Sixty percent had not experienced any new depressive episodes in the 3-year follow-up period, and the static relapse rates were low (11–15%). Recovery rates ranged from 69 to 97% depending upon the four different criteria used. Nevertheless, 26% had sought out treatment for depression or other psychological difficulties. Most patients (70%) had experienced negative life events in the follow-up period, but these events did not influence current depression severity. Return to work outcomes were encouraging, as eight out of 13 patients that had been on benefits were no longer receiving benefits. Life satisfaction ratings showed mean scores around 70 (on a 0–100 scale) and showed a moderate to strong negative correlation with depression severity. In conclusion, MCT appears to be promising with respect to long-term effect. Randomized controlled trials should investigate if the long-term effect of MCT surpasses that of other evidence-based treatments for depression.

## Introduction

Depression is predicted to become the leading cause of disease burden in 2030 (van Zoonen et al., 2014). In many cases, depression is a recurrent or chronic condition, and number of previous depressive episodes predict relapses (Kessing et al., 2004). An important question regarding the effectiveness of psychotherapy therefore concerns its long-term effect, especially given depression’s high rates of recurrence.

It is very important to improve treatment outcomes for depression, as less than one-third of patients recover at post-treatment, and relapse rates are estimated to be 50% after 2 years (Cuijpers, 2015). Given the modest initial response to treatment and high relapse rates after treatment, there is a need to develop treatments with higher immediate and sustained effects. Metacognitive therapy (MCT; Wells, 2009) could be a promising alternative. MCT is based on the self-regulatory executive function model (S-REF; Wells and Matthews, 1994, 1996). In this model, depression is understood as a consequence of perseverative thinking styles (especially rumination and worry) and other unhelpful self-regulation strategies. This style of thinking and behaving is called the cognitive attentional syndrome (CAS) and is controlled by positive and negative metacognitive beliefs, as well as maladaptive executive control of attentional processes (Wells and Matthews, 1994). The metacognitive model is supported by data from cross-sectional and longitudinal studies of the effects of rumination and metacognitions on depressive symptoms (e.g., Papageorgiou and Wells, 2009; Solem et al., 2016). Similarly, a meta-analysis indicated that rumination is a maladaptive emotion-regulation strategy across disorders, including depression (Aldao et al., 2010). Rumination seems to exacerbate negative mood and could trigger and prolong depression (e.g., Nolen-Hoeksema et al., 2008). MCT could therefore be beneficial for people with depression as suggested by several treatment studies with recovery rates ranging around 70–80% (e.g., Wells et al., 2009, 2012; Dammen et al., 2016; Hagen et al., 2017; Normann and Morina, 2018).

The current study is a follow-up of our randomized wait list controlled study that investigated the efficacy of MCT for depression (Hagen et al., 2017). Thirty-nine patients with depression were randomly assigned to immediate MCT (10 sessions) or a 10-week wait list period (WL). The WL group received 10 sessions of MCT after the waiting period. Results showed that 70–80% were recovered at post-treatment and 6-month follow-up (Hagen et al., 2017). The treatment was also associated with large reductions in interpersonal problems, even though MCT does not directly target such problems (Strand et al., 2018). At 1-year follow-up (intent-to-treat analyses), 34 of the 39 patients participated and 67% were classified as recovered, 10% improved, and 23% were unchanged (Hjemdal et al., 2019). Treatment response was associated with reductions in rumination, worry, and metacognitive beliefs as predicted by the metacognitive model.

Compared with traditional cognitive-behavioral therapy (CBT), MCT has a different perspective on the psychological factors that maintain depression. CBT is the recommended treatment for unipolar depression (Butler et al., 2006), but relapse rates have ranged from 40 to 60% within a period of 2 years (e.g., Hollon et al., 2006; Vittengl et al., 2007). In a recent CBT trial, those who were offered adjunctive CBT had fewer depressive symptoms and were more likely to fulfill criteria for remission (28 vs. 18%) at long-term follow-up (Wiles et al., 2016). However, as the authors noted, the trial also reminds us that pharmacotherapy and CBT do not provide a complete solution as the mean score on the Beck Depression Inventory (BDI) was 19 at 3–5 year follow-up.

There are also critical issues related to evaluation of long-term treatment effects for depression trials that need to be addressed (Vittengl et al., 2007). Several studies only report static time point assessments (assessment focusing on symptoms during the past month and not the entire follow-up period). This leaves gaps in assessment time during which relapses may have gone undetected (e.g., patients who relapse but then remit). Studies with assessment strategies leaving gaps have lower relapse rates, as do studies with shorter follow-up periods, and those using only instrument cut-off values for defining relapse (Vittengl et al., 2007). The authors also noted that very few studies reported on retreatment during the follow-up period. It is therefore important to address these topics in long-term follow-up studies.

At present, we know little about the effects of MCT on work outcomes and quality of life. This is an important area because the socioeconomic aspect of depression is relevant in assessing long-term recovery. Anxiety and depression are in fact the largest contributors to lost working years among mental disorders in Norway (Knudsen et al., 2012), and individuals given disability benefits for a mental disorder are younger than individuals given disability benefits for other reasons. Therefore, there has been a call that studies should aim to improve interventions for common mental disorders, with the aim to help patients return to work, and address how to maintain these effects over time (Øverland et al., 2018).

In the present study, we therefore report 3-year follow-up data for participants treated with MCT for depression. Therapy should ideally help patients develop adaptive coping mechanisms and enable them to deal with adverse life events, thereby preventing or delaying relapse or attenuating the severity and duration of future depressive episodes. This study is the first to investigate the 3-year effect of MCT for depression. To overcome important gaps in the current MCT outcome literature, we aimed to assess longer term outcomes in depression and a wider range of outcomes such as return to work and life satisfaction in addition to mechanism variables. Despite the recurrent nature of depression, our main hypothesis was that the majority of the treated patients would still be recovered at 3-year follow-up. This was based on high initial responder rates and documented modification of metacognitive beliefs in the previous trial (Hagen et al., 2017; Hjemdal et al., 2019) as well as the positive effects of MCT on recurrent and persistent depression in particular (Wells et al., 2009, 2012; Winter et al., 2019).

## Methods

### Participants and Procedure

The trial was registered at ClinicalTrials.gov (NCT01608399) and ethical approval was obtained from the Regional Medical Ethics Committee in Norway (ref.nr. 2011/1138).

To be included in the study, patients had to be 18+ years and fulfill DSM-IV criteria for primary unipolar depression (including both single episode and recurrent depression). Exclusion criteria were: psychosis, suicidality, post-traumatic stress disorder (PTSD), cluster A or B personality disorder, substance dependence, and patients could not receive other therapies during the intervention. Participants were consecutively referred to the project, which took place at a university outpatient clinic. In all, 105 people were interviewed for eligibility and 63% of these were excluded. Reasons for exclusions were primarily having other primary diagnoses. Eight had no diagnosis, five had subclinical depression, and two were excluded due to somatic disease. Included participants were randomly assigned to begin 10 sessions of MCT immediately or after a 10-week wait period with subsequent 10 sessions of MCT. For the current study, we combined the two treatment conditions as both groups had been treated with MCT.

Treatment followed the MCT manual for depression (Wells, 2009). Treatment consisted of case conceptualization and socialization to the model, learning to recognize rumination triggers, the Attention Training Technique, challenging beliefs about uncontrollability of rumination, challenging negative metacognitive beliefs about the danger and negative consequences of rumination, challenging positive metacognitive beliefs, eliminating maladaptive coping strategies (including avoidance), and finally relapse prevention including development of a therapy blue print.

Four therapists, all of whom were clinical psychologists trained in MCT (at the MCT-institute), delivered therapy. The originator of MCT (AW) supervised treatment based on videotaped recordings of the sessions and translated by bilingual therapists. In addition, the therapists met every second week for peer supervision. There were no therapist effects observed on BDI at 3-year follow-up, *F*(3, 33) = 1.24, *p* = 0.31. The assessment team consisted of two psychology graduate students trained in conducting clinical interviews.

### Measures

A diagnostic interview using the Structured Clinical Interview for the DSM-IV axis-I (SCID-I; First et al., 2002) was used to assess presence of axis-I disorders at follow-up. The assessment team did not use the entire interview, but selected parts of the interview based upon the patients’ diagnoses at pre-treatment. SCID-II interviews were not undertaken.

The assessment team also interviewed patients using the Hamilton Rating Scale for Depression (HRSD-17; Hamilton, 1967) to assess severity of depression. The HRSD-17 uses a 0 (absence of symptoms) to 4 (severe symptoms) scale. A total score between 0 and 6 indicates no depression, 7–17 mild depression, 18–24 moderate depression, and scores above 24 indicate severe depression.

The Beck Depression Inventory (BDI; Beck et al., 1961) is a 21-item self-report depression inventory. Each item is rated on a 0–3 scale. A BDI total score of 0–9 indicates no depression, 10–18 mild depression, 19–29 moderate depression, and 30–63 severe depression.

Return to work outcomes were also collected. The assessment team interviewed participants regarding their work status. Work status was coded accordingly: full-time work (100% full paid work), part-time work (less than 80% paid work), full-time student (100% studies), part-time student (taking less than 30 ECTS-credits per semester), and social benefits (unemployment or disability benefits). Patients that were taking part-time studies in addition to 100% work were coded as full-time work, and students working part-time along with their studies were coded as full-time students. One patient worked 80% by choice, which was coded as full-time work.

The Beck Anxiety Inventory (BAI; Beck and Steer, 1990) is a 21-item self-report inventory assessing anxiety symptoms. Each item is rated on a 0–3 scale. A BAI total score of 0–7 indicates minimal anxiety, 8–15 mild anxiety, 16–25 moderate anxiety, and 26–63 severe anxiety.

The Ruminative Response Scale (RRS; Nolen-Hoeksema and Morrow, 1991) was used to assess levels of depressive rumination. The RRS has 22 items rated on a 1–4 scale where higher scores indicate higher levels of rumination.

The Positive Beliefs about Rumination Scale (PBRS; Papageorgiou and Wells, 2001b) was used to assess beliefs concerning possible benefits of rumination. The PBRS has nine items using a 1–4 scale where higher scores indicate more positive beliefs about rumination.

The Negative Beliefs about Rumination Scale (NBRS; Papageorgiou and Wells, 2001a) was used to assess beliefs about uncontrollability and harm of rumination as well as its interpersonal consequences. The NBRS has 13 items using a 1–4 scale where higher scores indicate more negative beliefs about rumination.

The Metacognitions Questionnaire-30 (MCQ-30; Wells and Cartwright-Hatton, 2004) is a generic questionnaire used to assess dysfunctional metacognitions according to metacognitive theory. The MCQ-30 has 30 items using a 1–4 scale, with higher scores indicating more maladaptive metacognitions.

The Penn State Worry Questionnaire (PSWQ; Meyer et al., 1990) was used to assess worry. The PSWQ has 16 items rated on a 1–5 scale, with higher scores indicating higher levels of worry.

The Inventory of Interpersonal Problems (IIP-C; Alden et al., 1990) maps different types of interpersonal problems (domineering, vindictive, cold, socially avoidant, non-assertive, exploitable, nurturant, and intrusive). Example items from the IIP-C include “It is hard for me to join in on groups” and “I try to control others too much.” The items are scored on a 0 (not at all) to 4 (extremely) scale. The IIP-C total score represents a global score of interpersonal problems.

Negative life events: The assessment team interviewed patients about negative life events and asked open ended questions at first whether they had experienced any negative life events in the past 3 years. After the open-ended question, patients were asked specifically whether they had experienced any negative life event with respect to education/work, diseases, death of loved ones, relationships, judicial, and economy. Responses were coded dichotomously as having or having not experienced a significant negative life event as well as what type of life event it was.

Life satisfaction ratings: A set of items rated on a 0–100 scale was used to assess different aspects of quality of life. The assessment team interviewed patients by asking them how satisfied they were with their physical activity, diet, family functioning, social functioning, leisure time, and their view of their own future. In addition, the assessment team rated patients using a general assessment of functioning (GAF; both for symptoms and function) after finishing the interview.

### Statistical Analyses

Data were analyzed according to the intention to treat (ITT) protocol. There were five patients lacking follow-up data. These were replaced using the expectation-maximization method, except for return to work data, which used last observation carried forward for these five patients (post-treatment work status).

We report recovery rates based on several different criteria, as a range of criteria have been used in the literature: the first method for estimating recovery was based on Frank et al. (1991) where recovery was defined as no longer meeting diagnostic criteria for depression and scoring less than or equal to eight on the BDI. The second method for estimating recovery was based on the HRSD-17 and involved scoring seven or less on this interview. The third method for assessing recovery was based on Jacobson et al. (1999), with a cut-off point of 14 and reliable change index of 8.46 (i.e., a change of at least 9 points) on the BDI (Seggar et al., 2002). Patients were coded as improved if they only met one of these two criteria. The last method for assessing recovery was based on the 50% improvement (on BDI) criterion as used in some related studies (e.g., Wiles et al., 2016).

## Results

### Preliminary Analyses

Of the original 39 patients (of which two never started treatment) enrolled in the study, 31 attended the follow-up interview, three completed only self-report questionnaires, and we were unable to contact five of the patients. There were no significant differences in BDI scores at follow-up between patients who received MCT immediately and those who did so after a 10-week waiting list period, *t*(37) = 1.24, *p* = 0.22.

The eight people who did not attend the follow-up interview were compared with the 31 people who did. There were no significant differences in depressive symptoms between these two groups at pre-treatment (*p* = 0.69 and *p* = 0.75) or post-treatment (*p* = 0.33 and *p* = 0.63) as measured with the primary outcome variables (HRSD-17 and BDI respectively). Only BDI scores were available at 6-month and 1-year follow-up. There was no significant difference at 6-month follow-up, *t*(37) = 0.72, *p* = 0.47, nor at 1-year follow-up, *t*(37) = 1.22, *p* = 0.23, between these two groups.

There was no difference between sexes on BDI score at 3-year follow-up, *t*(37) = 1.23, *p* = 0.23 (nor at any other time of assessment). Furthermore, there was no significant correlation between age and BDI at 3-year follow-up, *r* = 0.06, *p* = 0.74 (nor at any other time of assessment).

### Changes in Symptoms and Beliefs

There were large reductions in symptoms and related metacognitive beliefs from pre-treatment to follow-up. The largest effect sizes were found for symptoms of depression and rumination. A summary of these changes is displayed in Table 1. The results showed reductions in symptoms of depression, anxiety, worry, rumination, negative beliefs about rumination, positive beliefs about rumination, interpersonal problems, and generic metacognitive beliefs. Results were stable from post-treatment to follow-up assessments.

**Table 1:** Changes in symptoms and beliefs from pre-treatment to 3-year follow-up (*N* = 39).

	Pre		Post		6-month		1-year		3-year		
	*M*	SD	*M*	SD	*M*	SD	*M*	SD	*M*	SD	*d*	
HRSD	19.92	3.58	5.33	6.54	n.a.		n.a.		4.97	4.66	3.60	
BDI	27.38	6.21	6.64	8.04	8.21	9.45	8.26	8.52	6.88	6.53	3.22	
BAI	20.92	9.25	4.85	7.22	7.00	9.57	7.21	9.20	6.50	6.18	2.26	
RRS	59.38	7.56	31.00	9.75	32.15	10.32	32.10	10.62	32.03	9.28	3.23	
IIP-C	1.62	0.43	0.87	0.64	0.89	0.60	0.87	0.54	0.85	0.55	1.56	
NBRS	28.77	6.03	18.03	4.63	17.79	4.37	17.49	4.64	17.47	3.94	2.22	
PBRS	20.21	6.53	11.03	3.70	11.51	4.03	11.54	4.42	11.06	3.24	1.78	
MCQ	2.26	0.34	1.47	0.35	1.47	0.37	1.44	0.36	1.42	0.28	2.70	
PSWQ	58.00	10.26	37.97	10.76	39.13	12.43	38.90	11.77	38.94	13.62	1.58	

*HRSD, Hamilton rating scale for depression-17; BDI, Beck depression inventory; BAI, Beck anxiety inventory; RRS, Rumination response scale; IIP-C, Inventory of interpersonal problems; NBRS, Negative beliefs about rumination scale; PBRS, Positive beliefs about rumination scale; MCQ, Metacognitions questionairre-30; PSWQ, Penn state worry questionnaire. Cohen’s d was calculated for pre-treatment to 3-year follow-up using pooled standard deviations*.

### Diagnoses

At pre-treatment, 79.5% had a recurrent depression diagnosis and 20.5% had a single depressive episode diagnosis. Comorbidity within the sample was as follows at pre-treatment: 16 patients had one additional axis-I disorder (10 had GAD, two panic disorder, one social phobia, one hypochondriasis, one trichotillomania, and one eating disorder not otherwise specified), one patient also had a second comorbid axis-I disorder (binge-eating disorder).

Patients’ pre-treatment diagnoses were re-assessed at 3-year follow-up. Only three patients met criteria for clinical diagnoses: one had a depression diagnosis and two had primary GAD. The patient diagnosed with depression (and presenting with a BDI score of 32) was one of the patients who never started MCT treatment but chose to start treatment at another clinic instead. This patient also had a secondary diagnosis of GAD. The two other patients were given GAD diagnoses and one of them had a secondary depression diagnosis (BDI score of 17). The patient who was given a GAD diagnosis only had not experienced any depressive episodes since treatment termination and had a BDI score of 10.

There was no significant difference in 3-year follow-up BDI scores for patients with and without comorbid disorders as assessed at pre-treatment, *t*(37) = 1.39, *p* = 0.17. Similarly, pre-treatment axis-II disorders (13 patients) were not related to poorer outcome at follow-up, *t*(37), = 1.70, *p* = 0.10. Axis-II disorders were not assessed at follow-up. We also investigated whether being diagnosed with recurrent depression (*n* = 33) at pre-treatment was associated with worse prognosis than having only one single episode (*n* = 6). Although BDI scores at follow-up were slightly higher for the recurrent depression group [7.5 (6.8) vs. 3.3 (3.2)], a repeated measures ANOVA with Greenhouse-Geisser correction did not find any significant time×condition effect (*p* = 0.17). All six patients with a single episode of depression were recovered, while for the recurrent group 27 were recovered, five improved, and one unchanged. Participants scoring above 30 on BDI (severe symptoms) at pre-treatment had lower likelihood of being recovered (Frank’s criteria) at 3-year follow-up, *χ*^2^ = 5.8, *p* = 0.016. However, this trend was not evident for patients scoring above 24 on the HRSD-17, *χ*^2^ = 0.40, *p* = 0.53.

### Recovery at 3-Year Follow-Up

Four different methods were used for assessing recovery rates. When using the Jacobson et al. (1999) method for clinically significant change, 84.6% were classified as recovered, 12.8% were improved, one patient showed no change, and none of the patients had deteriorated. When using the 50% improvement in symptoms criterion, 97.4% were classified as recovered. When using Frank et al. (1991) criteria, 69.2% were recovered, 28.2% were improved (no longer any depression diagnosis), while one patient was unchanged. The fourth method for assessing recovery was a score of seven or less on the HRSD-17. A total of 79.5% were classified as recovered using this method. In summary, recovery rates ranged from 69.2 to 97.4%, while the proportion of unchanged patients ranged from 2.6 to 20.5%. A summary of the different recovery rates is displayed in Table 2.

**Table 2:** Results of different methods for assessing recovery at follow-up.

	CSC	50% BDI reduction	BDI ≤ 8 + no diagnosis	HRSD ≤ 7
	*N* (%)	*N* (%)	*N* (%)	*N* (%)
Recovered	33 (84.6)	38 (97.4)	27 (69.2)	31 (79.5)
Improved	5 (12.8)	–	10 (25.6)	–
No change	1 (2.6)	1 (2.6)	2 (5.1)	8 (20.5)
Deteriorated	0	–	–	–

*CSC, Clinically significant change; BDI, Beck depression inventory; HRSD, Hamilton rating scale for depression. Recovered for the CSC category was defined as a score of 14 or less and a 9-point change on BDI, while the improved category was defined as 14 or less or a 9-point change on BDI. Frank’s criteria for improvement was defined as having no diagnosis of depression (but not meeting criteria for eight points or less on the BDI)*.

### Relapse Rates at 3-Year Follow-Up

We defined relapse in different ways. First, in relation to experiencing another depressive episode following treatment. We also examined the number of recovered patients no longer meeting criteria for recovery at 3-year follow-up.

The assessment team investigated depressive episodes since treatment termination in 30 of the patients. The majority of patients had not experienced any new depressive episodes (*n* = 18, 60%). However, five patients reported having experienced one depressive episode. Furthermore, three patients reported two episodes, two patients reported three episodes, and two patients reported four depressive episodes. Patients who had experienced new episodes had higher BDI scores at follow-up (10.7 [8.1] vs. 4.8 [5.3]) compared to patients without any new episodes, *t*(28) = 2.42, *p* = 0.02. There was also a non-significant trend toward a higher post-treatment score (9.9 [10.0] vs. 3.8 [5.7]) among patients who relapsed, *t*(28) = 1.93, *p* = 0.07.

We also investigated changes in recovery rates from post-treatment to 3-year follow-up. When using the Frank et al. (1991) criteria for recovery, 26 patients were recovered at post-treatment. Four of these 26 patients (15%) were no longer recovered at 3-year follow-up. Among the 13 patients that were not recovered at post-treatment, five were now recovered (38%), while eight were still not recovered. However, the majority of patients showed improvement as only two patients were classified as having no change.

When using the HRSD-17 criteria for recovery (scoring seven or below), a total of 28 patients were recovered at post-treatment. Three (11%) of these were not recovered at 3-year follow-up. Among the 11 patients that were not recovered at post-treatment, six (55%) were recovered at 3-year follow-up. In summary, the static relapse rates ranged from 11 to 15%. No suicides or suicide attempts were reported in the follow-up period.

### Return to Work Outcomes

At pre-treatment, 12 patients worked full time, eight were students (some of which had part-time jobs), six worked part time, while 13 were unemployed or received social or welfare benefits. At 3-year follow-up, 26 had full-time work, eight were full-time students, and five patients received benefits. Of the 13 patients who received benefits at pre-treatment, eight were now in full-time work. Patients receiving benefits at follow-up had higher BDI scores (*M* = 14.55, SD = 10.59) at follow-up than patients in full-time work (*M* = 5.91, SD = 5.07) and students (*M* = 5.23, SD = 5.20), *F*(36, 2) = 4.78, *p* = 0.014.

### Other Treatments in the Follow-Up Period

Of the 31 people who attended the interview, 23 (74%) had not received any psychotherapy since treatment termination. One patient had been in treatment for trichotillomania, one had two sessions of psychodrama, and six patients had been to sessions with a psychologist/psychiatrist. Patients who had sought out treatment had a higher BDI follow-up score (12.4 [8.4] vs. 5.6 [5.9]) compared to patients who had not been in additional therapy, *t*(29) = 2.48, *p* = 0.02. In fact, the re-treatment group also had higher BDI scores at pre-treatment (*p* = 0.03), post-treatment (*p* = 0.01), 6-month follow-up (*p* = 0.04), and 1-year follow-up (*p* = 0.02).

At pre-treatment, three patients were taking SSRIs, and nine had previously been treated with SSRIs. When assessed at follow-up, 25 reported that they had not used SSRIs, five had tried or were still using SSRIs in the follow-up period, and one had quit using anti-depressants after the trial. The five patients using SSRIs at follow-up had higher BDI scores (14.2 [11.7] vs. 6.1 [5.3]) compared to those not using such drugs, *t*(29) = 2.53, *p* = 0.02. These five patients had also been diagnosed with recurrent depression at pre-treatment.

### Negative Life Events Since Treatment Termination

Negative life events were also investigated in this sample. Nine people reported no serious negative life events during the 3-year period. However, 21 (70%) had experienced challenging events (some had multiple events): 13 had experienced serious illness/death in their family, one had financial difficulties, 10 had problems with their family (e.g., separated/divorced or family conflict), five had experienced conflicts at work, and one patient reported being subjected to a criminal offense. Patients experiencing negative life events did not report different BDI scores at follow-up compared to patients who reported no significant life events, *t*(28) = 0.82, *p* = 0.42, and number of negative life events was not associated with experiencing new depressive episodes, *t*(26) = 0.68, *p* = 0.50.

### Life Satisfaction Ratings

Patients’ self-reported quality of life was assessed using a range of subjective 0–100 scale questions (0 – very dissatisfied, 100 – very satisfied) and GAF-symptom and GAF-function ratings. They were asked how satisfied they were with their physical activity, diet, family, social life, leisure activities, and their view of the future. Most scores were in the 70’s, while physical activity had the lowest mean score of 62. A summary of these scores is provided in Table 3. These quality of life indicators showed a moderate to strong negative correlation with depression scores at follow-up.

**Table 3:** General assessment of functioning and life satisfaction ratings (0–100) and their relationship with depressive symptoms.

	*M*	SD	Min	Max	BDI *r*	HRSD *r*
**Assessment of functioning**						
GAF-symptoms	74.23	10.97	51	95	−0.76	−0.77
GAF-function	79.96	8.60	60	91	−0.80	−0.82
**Satisfaction ratings**						
Physical activity	62.03	20.19	0	100	−0.39	−0.35
Diet	74.53	12.10	40	100	−0.63	−0.45
Family	72.97	17.91	25	100	−0.48	−0.47
Social	72.03	21.08	0	100	−0.59	−0.49
Leisure activities	68.91	19.08	0	100	−0.37	−0.40
Optimism for the future	77.50	16.10	40	100	−0.56	−0.60

*GAF, Global assessment of functioning; HRSD, Hamilton rating scale for depression; BDI, Beck depression inventory. All items rated using a 0–100 scale. All correlations were significant, p < 0.01*.

## Discussion

The main aim of the current study was to explore symptom outcomes and general functioning 3 years after completing 10 sessions of MCT for depression. A total of 31 patients attended follow-up interviews, three provided only self-report data, while we were unable to contact five patients (*N* = 39). Therefore, we had complete data on 87% of all patients enrolled in the study. The patients not attending follow-up interviews were not different from those attending with respect to symptoms at post-treatment or short-term follow-up. This suggests that there is little indication of selection bias in the current study. The long-term results were encouraging. Only one patient fulfilled diagnostic criteria for major depression at 3-year follow-up while two were diagnosed with GAD. Symptom severity at 3-year follow-up was comparable for patients with and without comorbidity at pre-treatment. The majority of patients (60%) had not experienced any depressive episodes in the 3-year follow-up period, but 40% reported having experienced at least one new episode. There were large reductions in symptoms and beliefs since pre-treatment, and recovery rates ranged from 69 to 97% depending upon criteria used. Furthermore, static relapse rates were low (11–15%, *n* = 3–4) as defined by being classified as recovered at post-treatment but not at follow-up, and 38–55% (*n* = 5–6) of patients not recovered at post-treatment were recovered at 3-year follow-up. Patients may have learned adaptive beliefs and skills during treatment that helped them to overcome recurrent depressive episodes after treatment, as 70% had experienced negative life events during the 3-year follow-up period without detected deterioration or relapse.

The results showed large reductions not only in depressive symptoms, but also in anxiety, rumination/worry, interpersonal problems, and dysfunctional metacognitions that were maintained at 3-year follow-up. Also, treatment outcomes were similar for patients with and without comorbidity. This could suggest that MCT has high transdiagnostic utility. The positive treatment outcome was also supported by the return to work outcomes and quality of life ratings. Eight of 13 patients initially on benefits were no longer receiving benefits.

Similar studies that have examined the long-term effect of psychotherapy for depression have reported recovery rates at 18-month follow-up ranging from 19 to 30% (Shea et al., 1992), and relapse rates from 40 to 60% within a period of 2 years (Hollon et al., 2006; Vittengl et al., 2007). The meta-analysis of Vittengl et al. (2007) showed that relapse rates increased for studies with longer follow-ups and for studies not leaving gaps in the follow-up period. In the current study, post-treatment recovery rates were high (70–80%). Forty percent reported a new depressive episode during the follow-up phase and the static relapse rate was 11–15%. In a related SSRI/CBT trial, the mean score on BDI was 19 at 3–5 year follow-up (Wiles et al., 2016). In comparison, the mean BDI score in the current study was 6.9. This promising result warrants a replication of long-term effects in a randomized controlled trial comparing the reported long-term effects of MCT with other evidence-based therapies, as there are likely to be differences in sampling and recruitment across studies that challenge the validity of simple comparisons.

The meta-analysis by Vittengl et al. (2007) also highlighted the fact that few studies report incidents of retreatment. In the current study, 26% had sought out other treatments since completing the study and some for other problems than depression. A potential trigger for reoccurring depressive episodes could be negative life events and 70% of patients reported one or several negative life events following treatment. However, negative life events were not related to depression severity or relapse at follow-up. Another risk factor for relapse is the number of previous depressive episodes, but follow-up scores were not significantly different for patients who had recurrent depression compared to patients with only one single episode.

Some of the strengths of this study include detailed long-term assessment, high participation rate, and positive treatment outcomes. However, the study also suffers from limitations. Results could be attributed to other factors such as sample characteristics or therapist effects. Future randomized controlled studies should compare the MCT long-term effect with other evidence-based treatments for depression. Also, the assessment team only used part of the diagnostic interview based upon pre-treatment diagnoses. Using the entire interview, as well as SCID-II, could possibly have uncovered more diagnoses in the group, although the symptom- and life-satisfaction measures suggest that this was not the case. Another limitation of the study is that data were lacking on severity and length of new depressive episodes.

The present study has some important clinical implications. The study demonstrated that MCT for depression was associated with significant symptom reduction 3 years after treatment. Treatment was associated with reduction in the need for social benefits and an increase in work participation. The MCT protocol used in the study consisted of 10 treatment sessions, which is a relatively brief treatment and the results therefore indicate that this has the potential to be a cost-effective intervention. Future studies should therefore assess these parameters.

## Data Availability Statement

The datasets generated for this study are available on request to the corresponding author.

## Ethics Statement

All subjects gave a written informed consent in accordance with the Declaration of Helsinki. The trial was registered at ClinicalTrials.gov and approved by the Regional Medical Ethics Committee in Norway (ref.nr. 2011/1138). The study was not externally funded.

## Author Contributions

RH, OH, SS, and LK conducted the therapy in the trial. HN and AW provided clinical supervision. All authors have contributed in writing up the manuscript.

### Conflict of Interest

The authors declare that the research was conducted in the absence of any commercial or financial relationships that could be construed as a potential conflict of interest.

## References

[ref1] Aldao A. Nolen-Hoeksema S. Schweizer S. (2010). Emotion-regulation strategies across psychopathology: a meta-analytic review. Clin. Psychol. Rev. 30, 217–237. 10.1016/j.cpr.2009.11.004, PMID: 20015584

[ref2] Alden L. E. Wiggins J. S. Pincus A. L. (1990). Construction of circumplex scales for the inventory of interpersonal problems. J. Pers. Assess. 55, 521–536. 10.1207/s15327752jpa5503&4_10, PMID: 2280321

[ref3] Beck A. T. Steer R. A. (1990). Manual for the Beck anxiety inventory. San Antonio, TX: Psychological Corporation.

[ref5] Beck A. T. Ward C. H. Mendelson M. Mock J. Erbaugh J. (1961). An inventory for measuring depression. Arch. Gen. Psychiatry 4, 561–571. 10.1001/archpsyc.1961.01710120031004, PMID: 13688369

[ref6] Butler A. C. Chapman J. E. Forman E. M. Beck A. T. (2006). The empirical status of cognitive-behavioral therapy: a review of meta-analyses. Clin. Psychol. Rev. 26, 17–31. 10.1016/j.cpr.2005.07.003, PMID: 16199119

[ref7] Cuijpers P. (2015). Psychotherapies for adult depression: recent developments. Curr. Opin. Psychiatry 28, 24–29. 10.1097/YCO.0000000000000121, PMID: 25415495

[ref8] Dammen T. Papageorgiou C. Wells A. (2016). A two year follow up study of group metacognitive therapy for depression in Norway. J. Dep. Anxiety 5:227. 10.4172/2167-1044.1000227 25124119

[ref9] First M. B. Spitzer R. L. Gibbon M. Williams J. B. W. (2002). Structured clinical interview for DSM-IV-TR axis I disorders, research version, patient edition (SCID-I/P). New York, NY: Biometrics Research, New York State Psychiatric Institute.

[ref10] Frank E. Prien R. Jarrett R. B. Keller M. B. Kupfer D. J. Lavori P. W.. (1991). Conceptualization and rationale for consensus definitions of terms in major depressive disorder: remission, recovery, relapse and recurrence. Arch. Gen. Psychiatry 48, 851–855. 10.1001/archpsyc.1991.01810330075011, PMID: 1929776

[ref11] Hagen R. Hjemdal O. Solem S. Kennair L. E. O. Nordahl H. M. Fisher P.. (2017). Metacognitive therapy for depression in adults: a waiting list randomized controlled trial with six months follow-up. Front. Psychol. 8:31. 10.3389/fpsyg.2017.00031, PMID: 28174547 PMC5258745

[ref12] Hamilton M. (1967). Development of a rating scale for primary depressive illness. Br. J. Soc. Clin. Psychol. 6, 278–296. 10.1111/j.2044-8260.1967.tb00530.x, PMID: 6080235

[ref13] Hjemdal O. Solem S. Hagen R. Kennair L. E. O. Nordahl H. M. Wells A. (2019). A Randomized Controlled Trial of Metacognitive Therapy for Depression: Analysis of 1-Year Follow-Up. Front. Psychol. 10:1842. 10.3389/fpsyg.2019.01842 31440193 PMC6694776

[ref14] Hollon S. D. Stewart M. O. Strunk D. (2006). Enduring effects for cognitive behaviour therapy in the treatment of depression and anxiety. Annu. Rev. Psychol. 57, 285–315. 10.1146/annurev.psych.57.102904.190044, PMID: 16318597

[ref15] Jacobson N. S. Roberts L. J. Berns S. B. McGlinchey J. B. (1999). Methods for defining and determining the clinical significance of treatment effects: description, application, and alternatives. J. Consult. Clin. Psychol. 67, 300–307. 10.1037/0022-006X.67.3.300, PMID: 10369050

[ref16] Kessing L. V. Hansen M. G. Andersen P. K. Angst J. (2004). The predictive effect of episodes on the risk of recurrence in depressive and bipolar disorders - a life-long perspective. Acta Psychiatr. Scand. 109, 339–344. 10.1046/j.1600-0447.2003.00266.x, PMID: 15049770

[ref17] Knudsen A. K. Øverland S. Hotopf M. Mykletun A. (2012). Lost working years due to mental disorders: an analysis of the Norwegian disability pension registry. PLoS One 7:e42567. 10.1371/journal.pone.0042567, PMID: 22905150 PMC3419710

[ref18] Meyer T. J. Miller M. L. Metzger R. L. Borkovec T. D. (1990). Development and validation of the Penn state worry questionnaire. Behav. Res. Ther. 28, 487–495. 10.1016/0005-7967(90)90135-6, PMID: 2076086

[ref19] Nolen-Hoeksema S. Morrow J. (1991). A prospective study of depression and posttraumatic stress symptoms after a natural disaster: the 1989 Loma Prieta earthquake. J. Pers. Soc. Psychol. 61, 115–121. 10.1037/0022-3514.61.1.115, PMID: 1890582

[ref20] Nolen-Hoeksema S. Wisco B. E. Lyubomirsky S. (2008). Rethinking rumination. Perspect. Psychol. Sci. 3, 400–424. 10.1111/j.1745-6924.2008.00088.x, PMID: 26158958

[ref21] Normann N. Morina N. (2018). The efficacy of metacognitive therapy: a systematic review and meta-analysis. Front. Psychol. 9:2211. 10.3389/fpsyg.2018.02211, PMID: 30487770 PMC6246690

[ref22] Øverland S. Grasdal A. L. Reme S. E. (2018). Long-term effects on income and sickness benefits after work-focused cognitive-behavioural therapy and individual job support: a pragmatic, multicentre, randomised controlled trial. Occup. Environ. Med. 75, 703–708. 10.1136/oemed-2018-105137, PMID: 30032103

[ref23] Papageorgiou C. Wells A. (2001a). Metacognitive beliefs about rumination in recurrent major depression. Cogn. Behav. Pract. 8, 160–164. 10.1016/S1077-7229(01)80021-3

[ref24] Papageorgiou C. Wells A. (2001b). Positive beliefs about depressive rumination: development and preliminary validation of a self-report scale. Behav. Ther. 32, 13–26. 10.1016/S0005-7894(01)80041-1

[ref25] Papageorgiou C. Wells A. (2009). A prospective test of the clinical metacognitive model of rumination and depression. Int. J. Cogn. Ther. 2, 123–131. 10.1521/ijct.2009.2.2.123

[ref26] Seggar L. B. Lambert M. J. Hansen N. B. (2002). Assessing clinical significance: application to the beck depression inventory. Behav. Ther. 33, 253–269. 10.1016/S0005-7894(02)80028-4

[ref27] Shea T. Elkin I. Imber S. D. Sotsky S. M. Watkins J. T. Collins J. F.. (1992). Course of depressive symptoms over follow-up: findings from the National Institute of Mental Health treatment of depression collaborative research program. Arch. Gen. Psychiatry 49, 782–787. 10.1001/archpsyc.1992.01820100026006, PMID: 1417430

[ref28] Solem S. Hagen R. Hoksnes J. J. Hjemdal O. (2016). The metacognitive model of depression: an empirical test in a large Norwegian sample. Psychiatry Res. 242, 171–173. 10.1016/j.psychres.2016.05.056, PMID: 27285952

[ref30] Strand E. R. Hagen R. Hjemdal O. Kennair L. E. O. Solem S. (2018). Metacognitive therapy for depression reduces interpersonal problems: results from a randomized controlled trial. Front. Psychol. 9:1415. 10.3389/fpsyg.2018.01415, PMID: 30131749 PMC6090231

[ref31] van Zoonen K. Buntrock C. Ebert D. D. Smit F. Reynolds C. F. III Beekman A. T.. (2014). Preventing the onset of major depressive disorder: a meta-analytic review of psychological interventions. Int. J. Epidemiol. 43, 318–329. 10.1093/ije/dyt175, PMID: 24760873 PMC4023317

[ref32] Vittengl J. R. Clark L. A. Dunn T. W. Jarrett R. B. (2007). Reducing relapse and recurrence in unipolar depression: a comparative meta-analysis of cognitive-behavioral therapy’s effects. J. Consult. Clin. Psychol. 75, 475–488. 10.1037/0022-006X.75.3.475, PMID: 17563164 PMC2630051

[ref33] Wells A. (2009). Metacognitive therapy for anxiety and depression. New York, NY: The Guilford Press.

[ref34] Wells A. Cartwright-Hatton S. (2004). A short form of the metacognitions questionnaire: properties of the MCQ-30. Behav. Res. Ther. 42, 385–396. 10.1016/S0005-7967(03)00147-5, PMID: 14998733

[ref35] Wells A. Fisher P. Myers S. Wheatley J. Patel T. Brewin C. R. (2009). Metacognitive therapy in recurrent and persistent depression: a multiple-baseline study of a new treatment. Cogn. Ther. Res. 33, 291–300. 10.1007/s10608-007-9178-2

[ref36] Wells A. Fisher P. Myers S. Wheatley J. Patel T. Brewin C. R. (2012). Metacognitive therapy in treatment resistant depression: a platform trial. Behav. Res. Ther. 50, 367–373. 10.1016/j.brat.2012.02.004, PMID: 22498310

[ref37] Wells A. Matthews G. (1994). Attention and emotion: A clinical perspective. Hove: Erlbaum.

[ref38] Wells A. Matthews G. (1996). Modelling cognition in emotional disorder: the S-REF model. Behav. Res. Ther. 34, 881–888. 10.1016/S0005-7967(96)00050-2, PMID: 8990539

[ref39] Wiles N. J. Thomas L. Turner N. Garfield K. Kounali D. Campbell J.. (2016). Long-term effectiveness and cost-effectiveness of cognitive behavioural therapy as an adjunct to pharmacotherapy for treatment-resistant depression in primary care: follow-up of the CoBalT randomised controlled trial. Lancet Psychiatry 3, 137–144. 10.1016/S2215-0366(15)00495-2, PMID: 26777773

[ref40] Winter L. Gottschalk J. Nielsen J. Wells A. Schweiger U. Kahl K. G. (2019). A comparison of metacognitive therapy in current versus persistent depressive disorder – a pilot outpatient study. Front. Psychol. 10:1714. 10.3389/fpsyg.2019.01714, PMID: 31447722 PMC6691034

